# Supplementary figures and images for: Heart rate variability in hypothyroid patients: A systematic review and meta-analysis
Source: PLoS One. 2022 Jun 3;17(6):e0269277. doi: 10.1371/journal.pone.0269277 (PMC9165841; doi:10.1371/journal.pone.0269277)

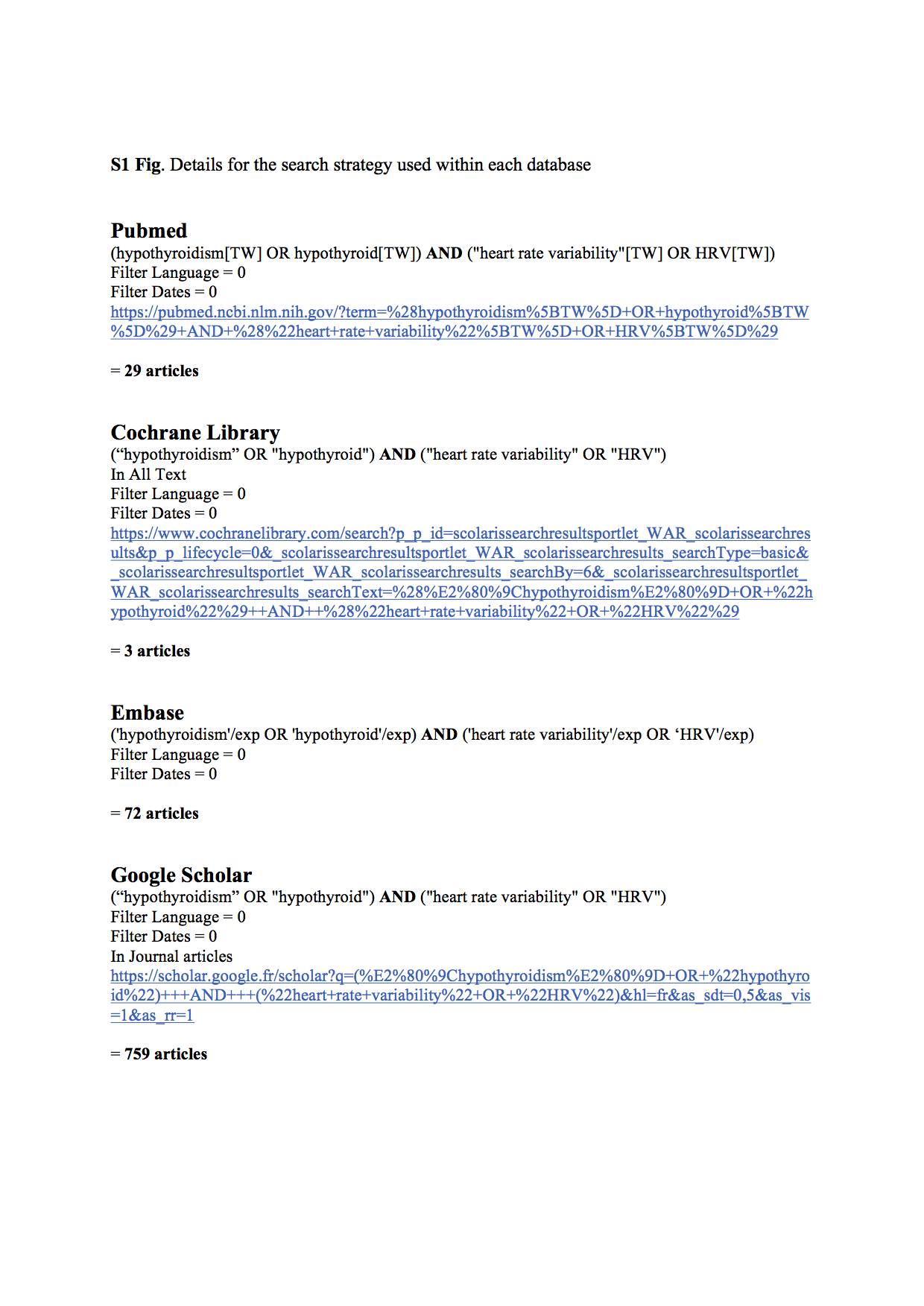

Supplement: S1 Fig — (TIFF) [file pone.0269277.s002.tiff]

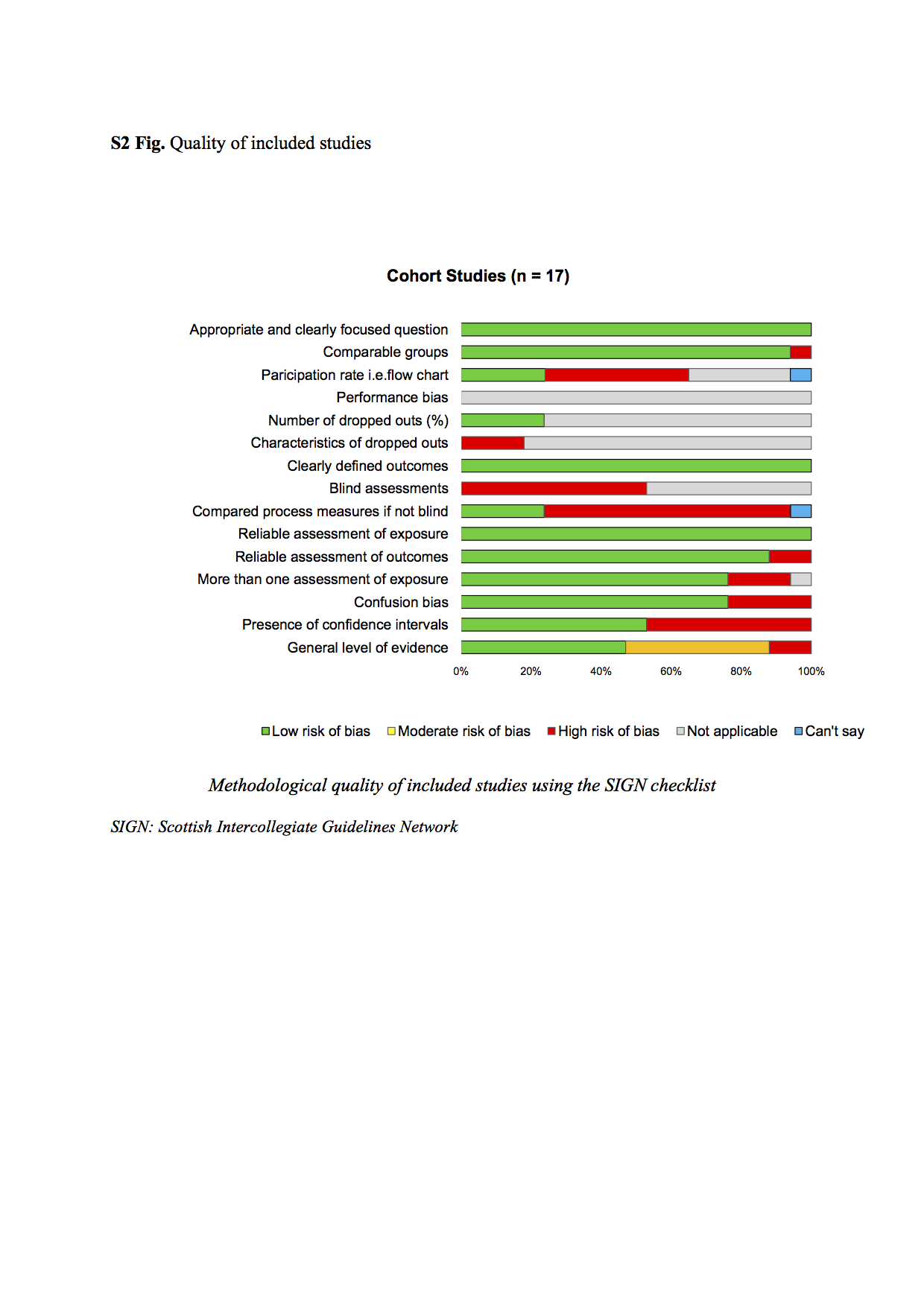

Supplement: S2 Fig — Methodological quality of included studies using the SIGN checklist. Methodological quality of included studies using the SIGN checklist, by study. SIGN checklist for cohort studies. Methodological quality of included studies using STROBE checklist, by study. (TIFF) [file pone.0269277.s003.tiff]

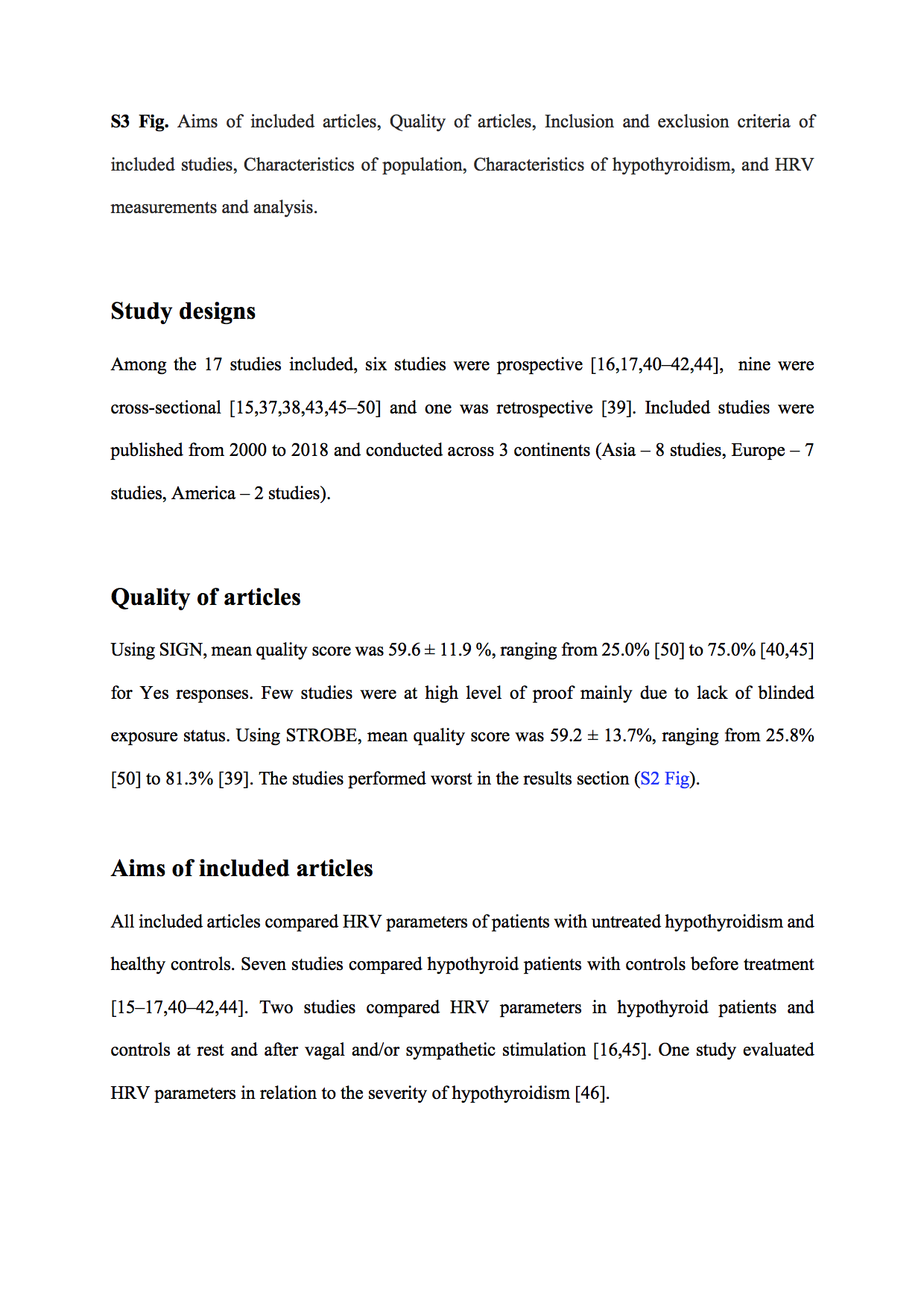

Supplement: S3 Fig — (TIFF) [file pone.0269277.s004.tiff]

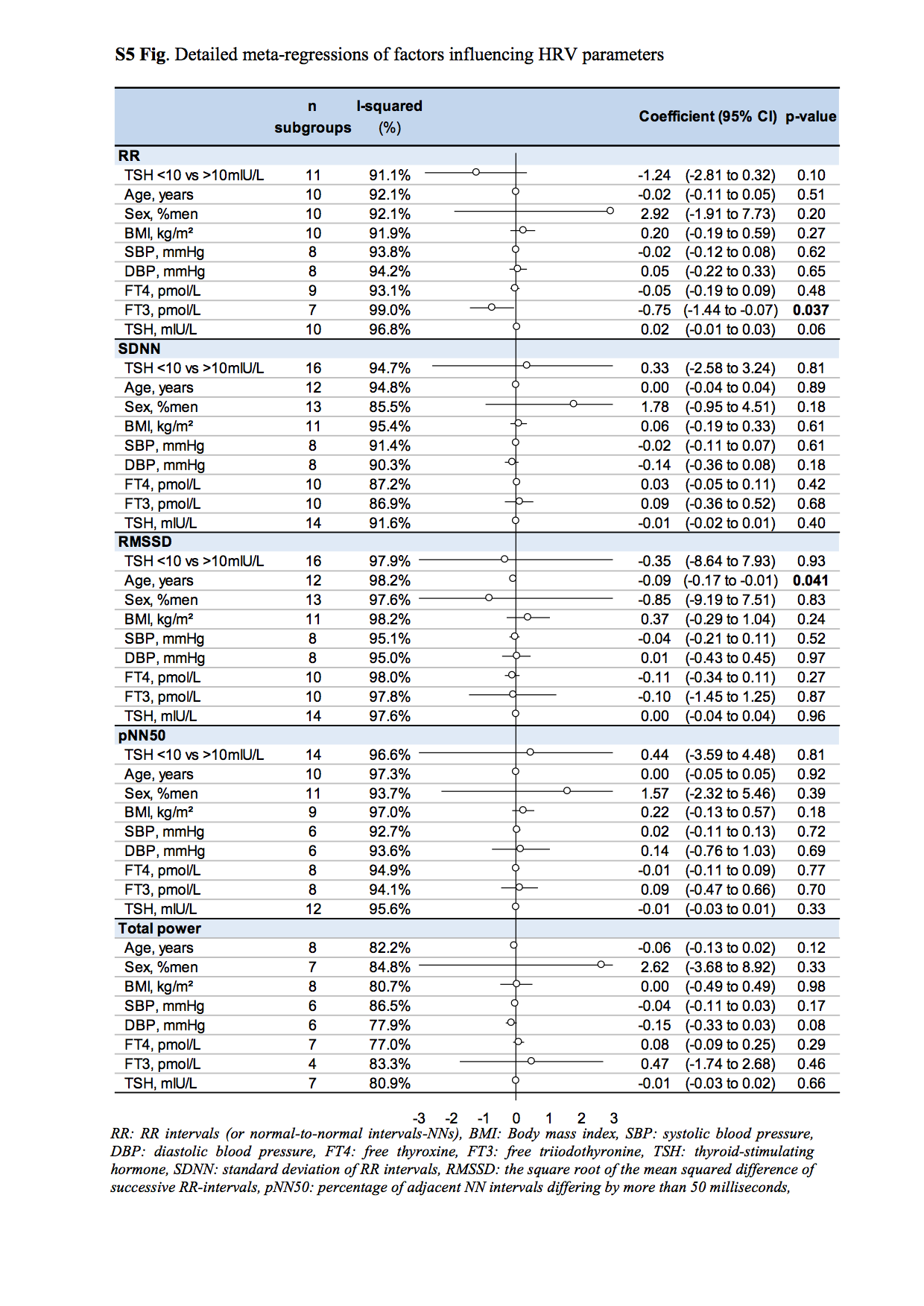

Supplement: S5 Fig — (TIFF) [file pone.0269277.s006.tiff]

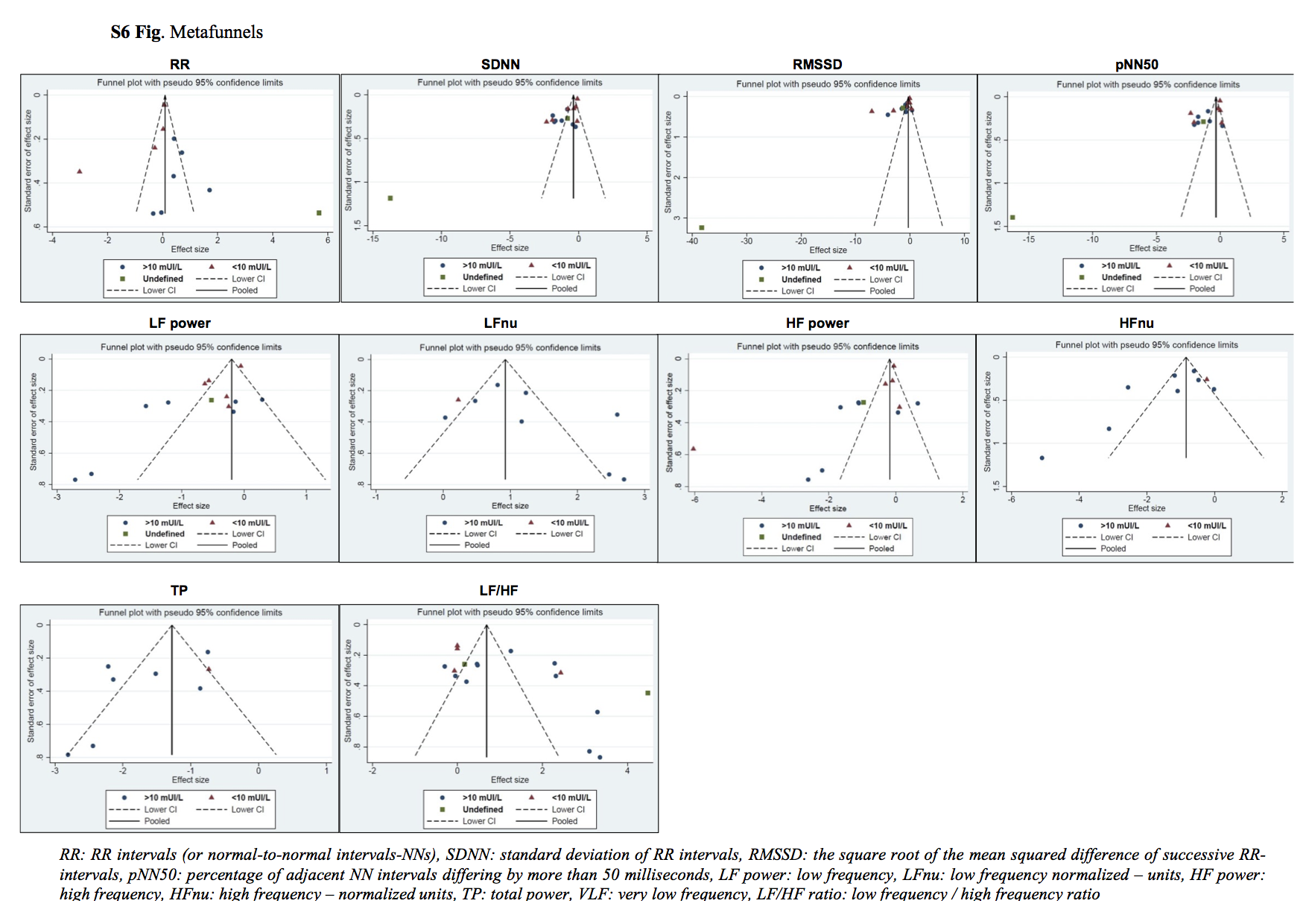

Supplement: S6 Fig — (TIFF) [file pone.0269277.s007.tiff]
